# Perceived Need and Acceptability of an App to Support Activities of Daily Living in People With Cognitive Impairment and Their Carers: Pilot Survey Study

**DOI:** 10.2196/16928

**Published:** 2020-07-31

**Authors:** Rhoda Lai, Maria Tensil, Alexander Kurz, Nicola T Lautenschlager, Janine Diehl-Schmid

**Affiliations:** 1 Academic Unit for Psychiatry of Old Age Department of Psychiatry The University of Melbourne Melbourne Australia; 2 Department of Psychiatry and Psychotherapy School of Medicine Technical University of Munich Munich Germany; 3 Aged Persons Mental Health Program North Western Mental Health Melbourne Health Melbourne Australia; 4 School of Psychiatry and Clinical Neurosciences & WA Centre for Health and Ageing University of Western Australia Perth Australia

**Keywords:** Aged, dementia, memory disorders, carers, mobile apps

## Abstract

**Background:**

Modern technologies, including smartphone apps, have the potential to assist people with cognitive impairment with activities of daily living, allowing them to maintain their independence and reduce carer burden. However, such tools have seen a slow rate of uptake in this population, and data on the acceptability of assistive technologies in this population are limited.

**Objective:**

This pilot study included older adults with cognitive impairment and their carers, and explored the perceived needs for and acceptability of an app that was designed to be a simple assistive tool for activities of daily living. In particular, this study aimed to assess the acceptability of common app functions such as communication, reminder, navigation, and emergency tools in this population, and to compare patients’ and carers’ responses to them.

**Methods:**

A total of 24 German participants with mild cognitive impairment or dementia and their family carers separately completed two short questionnaires. The first questionnaire asked the participants with cognitive impairment and their carers to self-rate the patients’ cognitive impairment levels and affinity to technology. Following a demonstration of the app, participants rated the usability and acceptability of the app and its functions in a second questionnaire.

**Results:**

Participants rated themselves as much less cognitively impaired than their carers did (*P*=.01), and insight into the level of support they received was low. The majority of the participants (19/24, 79%) and their carers (20/24, 83%) had low affinity to technology, and even after the demonstration, 63% (15/24) of the participants had low interest in using the app. A breakdown of acceptability responses by app function revealed that participants were more amenable to the reminder function, the emergency feature, and a wearable form of the app. Features that centered around carers monitoring participants’ movements were reported to be less acceptable to participants.

**Conclusions:**

This study highlights the importance of focusing on acceptability and the consumer’s perceptions in the development of assistive technology for older adults with cognitive impairment. Participants showed an aversion to functions they perceived as eroding their independence, while functions that more closely aligned with independence and autonomy were perceived as more acceptable.

## Introduction

By 2050, the World Alzheimer's Report 2015 projected that over 20% of the world’s population will be over the age of 60 years [1]. With this comes a projected rise in the prevalence of dementia and mild cognitive impairment (MCI) [1]. The greatest cost of dementia care has been found to be unpaid or informal care, which costs 16,473€ (US $21,011) per patient annually in Germany, in addition to productivity losses for caregivers who are employed [2]. Most informal carers of people with memory problems are their spouses, who provide help with daily activities [2].

In recent years, there has been an increased interest in how modern technology can assist with the daily activities of older adults with cognitive impairment [3,4]. Technologies that are being explored include reminder systems (such as medication prompts), tools for maintaining social contact, navigational and safety monitoring tools, and stimulation tools (for entertainment or cognitive training) [3]. There is evidence to suggest that these technologies have the potential to not only benefit people with cognitive impairment by increasing or preserving their independence and safety, but also to subsequently support carers by reducing the time and energy spent on daily caregiving activities and reducing anxiety about taking respite time [4,5].

However, technologies for people with cognitive impairment have seen a slow rate of uptake, which could be partly attributed to a lack of user-centered design and validation [6,7]. Studies have tended to focus on technological possibilities and design rather than the usability and acceptability of these technologies for older people [8], which are particularly important to evaluate for this population that is often reluctant to adopt new technologies [9]. Even in studies that have evaluated technology usability among people with cognitive impairment, carers have often been the participants [10]—few have evaluated usability or acceptability for people with memory problems [9,11,12]. Furthermore, in the studies that have included people with memory problems, their opinions have not been reported separately from their carers’, which may differ significantly [9,12]. In order to be acceptable, the technology must not only be easy to use (ie, it must have high usability) but must also spark motivation in the user to adopt it in everyday life [9]. Little is known about which features people with cognitive impairment would value in assistive technology, and which would increase their motivation for using it. These insights may be key to increasing uptake rates in this population.

In this pilot study, we engaged in a preliminary exploration of the acceptability of a simple smartphone app among people with mild cognitive impairment and dementia and their family carers living in the community. The app was designed to aid the person with cognitive impairment in activities of daily living, including communicating with friends and family, navigating, and serving as a memory prompt and emergency alert system. The study aimed to obtain feedback from people with cognitive impairment and their family carers separately on their perceived need for such a support, and whether its functions would be useful and feasible for everyday use. Although we explored the usability and acceptability of each of the app’s functions, the focus of this study was not on the design of this app specifically, but rather on the participants’ and carers’ perceived need and usability of an assistive app in general.

## Methods

### The App

The app used was a pilot developed in collaboration with professionals in the fields of informatics and dementia. It used a simple interface and had four key functions: (1) simplified phone calls and voice or text messages to predefined contacts; (2) a memory aid, used to save speech and text notes as reminders, such as reminders for taking medications; (3) a one-click emergency call to the predefined carer; and (4) a simplified pedestrian navigation system. The app interface was in German.

### Participants and Recruitment

From an outpatient memory clinic in Germany, 24 older adults with a diagnosis of MCI or dementia and their family or informal carers were recruited (demographic data are displayed in Table 1). Patients were invited to participate in the study if (1) they had been diagnosed with MCI or mild dementia at a prior visit, and (2) if they had a close relative/carer. All patients who visited the memory clinic (irrespective of the reason, whether it was for a follow-up visit, clinical trial participation, or counseling) and fulfilled the inclusion criteria were asked to participate, and if they agreed, an appointment for the study visit was scheduled. Demographic data for the participants are displayed in Table 1. Of the 24 patients recruited, 14 (58%) of the patients lived in the same household as their carer.

**Table 1:** Participant demographic data.

Characteristic		Patients (N=24)	Carers (N=24)
Age (years), mean (SD, range)		74.5 (6.1, 57-84)	62.4 (16.0, 31-83)
**Gender, n (%)**			
	Men	8 (33)	15 (62)
	Women	16 (67)	9 (38)
Education, > 12 years, n (%)		8 (33)	11 (46)
MMSE^a^ score, mean (SD, range)		22.4 (4.2, 10-28)	
**Diagnosis, n (%)**			
	MCI^b^	6 (25)	N/A^c^
	Dementia caused by Alzheimer’s disease	16 (67)	N/A
	Other neurodegenerative dementia	2 (8)	N/A
**Relationship to patient, n (%)**			
	Spouse	N/A	7 (29)
	Child	N/A	7 (29)
	Other type of relative	N/A	1 (4)
	Friend	N/A	2 (8)

^a^MMSE: Mini-Mental State Examination.

^b^MCI: mild cognitive impairment.

^c^N/A: not applicable.

### Procedure and Measures

Patients and carers first completed a short, simple questionnaire in separate face-to-face interviews conducted in their native German. They each self-rated the patient’s impairments and obstacles in daily living by responding to questions about how often appointments and medications are forgotten, how often memory aids are used, and how often they become disoriented outside the home. Responses were indicated using “rarely,” “sometimes,” “often,” or “supported by carer” options, with the latter indicating that a task was perceived as possible only with carer support. They also each rated the patient’s affinity to technology and answered questions about the patient’s experience with using devices such as smartphones and computers. Demographic information was also collected in this questionnaire.

The interviewer then demonstrated the app to the patients and their carers, explaining all functions and answering any questions. Patients and carers were given the opportunity to try using the app’s functions, with help from the interviewer if needed. This lasted for approximately one hour.

Finally, patients and carers rated the usability and acceptability of the app. This was assessed through a short questionnaire administered in separate face-to-face interviews about the clarity of the layout, interest in finding out more about the app, interest in trying the app, and ratings of each function’s usefulness. Ratings of usability and acceptability were measured using “low,” “moderate,” “high,” or “don’t know” responses.

This project was approved by the Ethics Committee of the Medical Faculty of the Technical University of Munich (335/18S).

### Data Analysis

Data analysis was performed using the Statistical Package for the Social Sciences (SPSS; IBM Corp). Descriptive analyses were performed to summarize the socio-demographic data and frequencies generated for survey answers. A Wilcoxon signed-rank test was used to analyze differences between patients’ and carers’ perceptions of impairments in daily living. A statistical threshold of *P*<.05 was considered statistically significant.

## Results

The patients tended to rate their impairments as less severe than their carers did (see Table 2). When asked how often they had problems with aspects of their memory or concentration, carers responded that patients were affected more often than the patients did. The Wilcoxon signed-rank test revealed that perceptions differed significantly between patients and carers on every aspect of daily living that was asked about in the survey (see Table 2).

**Table 2:** Participants’ (N=24) self-rating, carers’ (N=24) rating of the participants’ impairments, and *P* values for differences between their perceptions.

Question		Response								
		Rarely		Sometimes	Often		Carer supported		*P* value	
**How often is the patient’s memory and concentration impaired?**										.012^a^
	Patients, n (%)	5 (21)	14 (58)			5 (21)		N/A^b^		
	Carers, n (%)	2 (8)	7 (29)			15 (63)		N/A^b^		
**How often does the patient forget appointments?**										.001^a^
	Patients, n (%)	12 (50)	6 (25)			2 (8)		4 (17)		
	Carers, n (%)	5 (21)	1 (4)			3 (13)		15 (62)		
**How often does the patient forget to take their medication?**										.007^a^
	Patients, n (%)	10 (42)	3 (12)			0 (0)		11 (46)		
	Carers, n (%)	3 (13)	2 (8)			2 (8)		17 (71)		
**How often does the patient get disoriented outside the home?**										.028^a^
	Patients, n (%)	17 (71)	3 (12)			0 (0)		4 (17)		
	Carers, n (%)	11 (46)	5 (21)			2 (8)		6 (25)		

^a^A statistical threshold of *P*<.05 was considered statistically significant.

^b^N/A: not applicable.

Of the 24 carers, 20 (83%) rated their own general affinity to technology as “low.” Despite this, half of the 24 carers reported that they had “high” (7/24, 29%) or “very high” (5/24, 21%) levels of preparedness for using an app, indicating their interest in using such an assistive tool. Half (12/24, 50%) of the 24 patients never used a mobile phone, and out of those who did, only 3 (13%) used a mobile phone at least once a week. Of the 24 carers, 19 (79%) reported that the patient’s ability to successfully use an app would be “low,” while the remaining 5 carers believed it would be “moderate.” In the predemonstration interview, most patients (18/24, 75%) reported “low” interest in getting a short introduction to the app and its use, which corresponded with the 19 (19/24, 79%) carers who believed their relative’s willingness and ability to use an app would be “low.” These responses were given despite the fact that 10 (42%) of the 24 patients reported frequent use of aids like calendars or notebooks for everyday tasks (while 4 patients, 17%, reported using such aids “occasionally”).

After the demonstration of the app, patients’ willingness to try the app only changed moderately—15 (63%) of the 24 patients still reported “low” interest in trying the app despite most of them (18/24, 75%) reporting that the layout was “clear.” However, their perceptions about the usefulness of each function varied (see Table 3). Patients rated the reminder and emergency functions more highly than the navigation function. Interestingly, carers were more likely to feel safe if they could see the patient’s location, whereas patients did not rate the usefulness of the navigation and location function highly. Surprisingly, a large proportion of patients thought that having the app in a wearable form, such as on a smartwatch, would be useful—7 (29%) of the 24 patients reported “moderate” or higher usefulness.

**Table 3:** Patients’ (N=24) and carers’ (N=24) ratings of the usefulness of the app’s functions.

Question		Response						
		Low	Moderate		High		Don’t know	
**How likely is it that the patient would use the navigation function?**								
	Patients, n (%)	14 (58)		1 (4)		1 (4)		8 (33)
	Carers, n (%)	16 (67)		8 (33)		0 (0)		0 (0)
**How much would it increase your feeling of safety if the carer could see the patient’s location?**								
	Patients, n (%)	16 (67)		2 (8)		2 (8)		4 (17)
	Carers, n (%)	13 (54)		7 (29)		4 (17)		0 (0)
**How likely is it that the patient would use the reminder function for appointments/medications?**								
	Patients, n (%)	9 (38)		7 (29)		2 (8)		6 (25)
	Carers, n (%)	13 (54)		4 (17)		7 (29)		0 (0)
**How useful would it be if the carer could update appointments in the app via the internet?**								
	Patients, n (%)	12 (50)		4 (17)		4 (17)		4 (17)
	Carers, n (%)	15 (62)		5 (21)		4 (17)		0 (0)
**How likely is it that the emergency function would be useful?**								
	Patients, n (%)	5 (21)		7 (29)		5 (21)		7 (29)
	Carers, n (%)	10 (42)		6 (25)		8 (33)		0 (0)
**How useful would it be if the app came in a wearable form, like on a smartwatch?**								
	Patients, n (%)	8 (33)		2 (8)		5 (21)		9 (38)
	Carers, n (%)	6 (25)		11 (46)		7 (29)		0 (0)

## Discussion

### Principal Findings

This pilot study included several findings that will be useful in the design of apps for people with cognitive impairment, including the significant discrepancy between perceived levels of cognitive impairment between people with cognitive impairment and their carers. There was low overall interest in such an app, and specifically, patients were least interested in functions that facilitated carers with monitoring their activities. This study has begun to address a gap in the research, surveying not only carers’ perceptions of assistive technology but also the perceptions of people with cognitive impairment.

In this study, there were clear discrepancies between the ratings given by the participants with cognitive impairment and their carers in every aspect of cognition. Participants with cognitive impairment most frequently responded that they rarely or only sometimes forgot appointments or became disoriented outside the home, and did not acknowledge that they were supported by their carers as often as carers reported they were. Limited insight into their own need for support in daily activities may be a barrier to the uptake of assistive technology by this population [6].

Even after receiving the demonstration of the app, almost two-thirds (15/24, 63%) of the participants with cognitive impairment had low-interest levels in trying the app, and they frequently responded with the “don’t know” option when rating the app functions’ usefulness, showing ambivalence toward adopting the technology. Previous research has shown that the use of technology can ignite positive feelings of mastery in older adults [12], and a review of mobile assistive technologies for people with dementia found that there was a dire need for these tools to tap into higher-level human needs, such as self-esteem and creativity [13]. However, the low-interest levels in this study point to a barrier to technology adoption. Studies have found that learning to use new technologies can call an older adult’s attention to their diminishing memory and functioning [14], and it is possible that participants in this study were responding to such a recognition with denial of their need for assistive apps. It is clear from this study that simply demonstrating the functionality of assistive technologies is not enough to spark interest in their use, and that it may even turn potential consumers away if their use is perceived to be demeaning or to highlight impairments [12].

Although this study does not offer a promising likelihood of people with cognitive impairment adopting such an app, it did provide useful findings on the acceptability of the app’s different functions. Most evident was the fact that people with cognitive impairment appeared to have a desire to maintain their independence. While participants with cognitive impairment were moderately open to the usefulness of the reminder function, they were less open to the idea of their carer being able to update their appointment information remotely. This desire also manifested in responses to the app’s navigation function—nearly half (11/24, 46%) of the carers reported that being able to see the patient’s location would at least moderately increase their feelings of safety, but about two-thirds (16/24, 67%) of the patients reported that it would not make them feel safer. The patients were more amenable to having an emergency function handy, which suggests that they preferred carer help to be on an on-call basis rather than as an ongoing presence. This view differed from the typical view of the carers, who leaned toward being supportive of surveillance and monitoring technology [12,15]; this should be noted in future studies.

Interestingly, in a qualitative study, Hill et al [16] found that older adults perceived technology to be contributing to the erosion of independence and skills such as problem-solving in younger people; these older adults correlated the ability to live without technology dependence with self-reliance and the preservation of cognitive skills [16]. Despite 42% (10/24) of the participants with cognitive impairment in this study reporting the use of aids to assist with everyday memory tasks, it is possible that participants could have been similarly resistant to using technological aids for the same reason. Further, in another qualitative study by Arntzen et al [17], people with younger onset dementia reported that new technology had to fit well into their habitual routines to facilitate adoption. The relative similarity between a smartwatch and a regular watch may bridge the adoption of a new technology, and this is supported by the finding that 7 out of 24 patients (29%) thought it would be useful to have the app on a smartwatch, a greater proportion of patient agreement than was observed for any other app function rating. Although this is mere speculation, the need to relate assistive technology to higher-level needs in order to facilitate better technology adoption rates is apparent [13]—the need for autonomy and identity maintenance was clear in this study’s population sample [17].

### Limitations and Future Directions

This study had several limitations. Participants were given limited time to trial the app; had they been permitted to trial the app at home, it is possible that they could have developed greater confidence in and acceptability of using the app over time. Furthermore, we did not capture in the questionnaire whether the participants completely understood the functionality of the app (although anecdotal impressions from the interviewer were that they grasped the concept). The study’s sample size was small, which limited the ability to conduct statistical analyses on the data.

However, this study captures attitudes that would be present in the decision-making moments regarding the use or purchase of an app. Future studies would benefit from involving people with cognitive impairment in all aspects of assistive technology development. While this population may find technologies acceptable when scaffolded by training and provision of devices, or in the evaluation phase, gauging interest at the outset may be vital to unpacking higher-level needs that increase technology use in this population [18].

### Conclusions

Overall, this study has provided useful preliminary findings on older adults with cognitive impairment and their carers’ perceptions of the usefulness of different functions that can be provided within an app to assist with everyday tasks. The need for assistive apps and technology to be tailored toward autonomy and identity maintenance to be acceptable was apparent. Aligning assistive technology to these needs may improve the uptake of technology in this population.

## Abbreviations

MCI: mild cognitive impairment
MMSE: Mini-Mental State Examination
SPSS: Statistical Package for the Social Sciences
